# Multimorbidity increases risk of atrial fibrillation recurrence after cryoballoon ablation

**DOI:** 10.1016/j.ijcha.2025.101700

**Published:** 2025-05-09

**Authors:** Marieke J.H. Velt, Colinda van Deutekom, Michelle Lobeek, Michiel Rienstra, Yuri Blaauw, Bart A. Mulder

**Affiliations:** a Department of Cardiology, University of Groningen, University Medical Center Groningen, Groningen, the Netherlands

**Keywords:** Multimorbidity, Comorbidities, Cryoballoon ablation, Recurrence

## Abstract

**Background and Aims:**

Despite advancements in atrial fibrillation (AF) catheter ablation techniques, atrial arrhythmia recurrence after the procedure remains common. Although individual comorbidities are known to affect ablation outcomes, the role of multimorbidity is unclear. This study aimed to investigate the association between multimorbidity and atrial arrhythmia recurrence after cryoballoon AF ablation.

**Methods:**

The cryoballoon ablation study is a single-center, prospective registry including 349 consecutive patients undergoing cryoballoon AF ablation. The presence of eleven comorbidities was assessed and the population divided into two groups: no multimorbidity (0–1 comorbidity) and multimorbidity (≥2 comorbidities). Clinical follow-up visits combined with 12-lead ECG and 24-hour Holter monitoring were scheduled at 3, 6 and 12 months post-procedure. Cox proportional hazard regression analyses were conducted to assess the association with atrial arrhythmia recurrence. Kaplan-Meier estimates for the cumulative risk of the first recurrence were calculated and plotted.

**Results:**

The mean age was 62 ± 9 years and 123 (35 %) were women. Multimorbidity was present in 184 (53 %) patients. During a median follow-up of 328 [IQR 203–409] days, 114 patients (33 %) experienced atrial arrythmia recurrence within one year after cryoballoon AF ablation. Cox proportional hazard regression analyses, adjusted for age and sex, revealed a significant association between multimorbidity (HR 1.64, 95 % CI 1.12–2.39) and atrial arrhythmia recurrence. Furthermore, hypertension (HR 1.58, 95 % CI 1.01–2.49) and obesity (HR 1.63, 95 % CI 1.09–2.42) were associated with recurrence at one year post-ablation.

**Conclusion:**

In AF patients undergoing cryoballoon AF ablation, multimorbidity was associated with atrial arrhythmia recurrence within one year post-ablation.

## Graphical abstract

**Figure:**
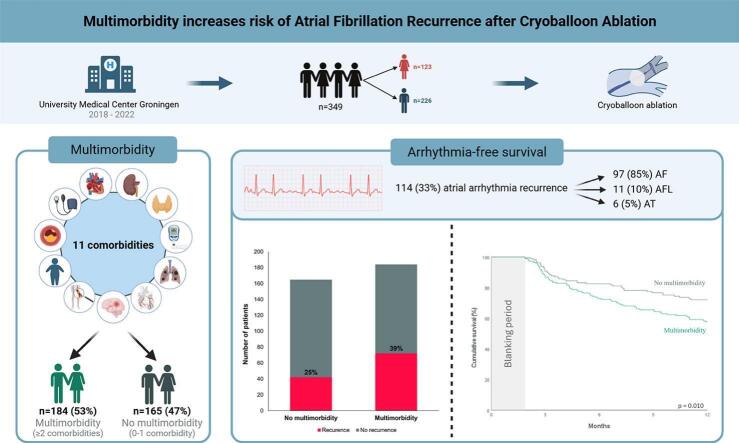

## 1 Introduction

Atrial fibrillation (AF) is the most common cardiac arrhythmia in adults with increasing incidence and prevalence [1]. Over the last decades, substantial advancements have been made in AF catheter ablation techniques, where pulmonary vein isolation (PVI) remains the cornerstone. The guidelines recommend catheter ablation for patients with paroxysmal or persistent AF, or when antiarrhythmic drugs are ineffective or poorly tolerated, as it significantly reduces AF burden and improves quality of life. [2] Despite the advancements in catheter ablation techniques, recurrence of atrial arrhythmias after the procedure remains of clinical concern [3].

AF rarely occurs in isolation, often presenting alongside comorbidities such as hypertension, heart failure, diabetes mellitus, obesity, and sleep apnea [4]. Each of these comorbidities has shown to negatively affect outcomes after ablation, increasing the likelihood of atrial arrhythmia recurrence [[5], [6], [7], [8], [9], [10], [11], [12], [13], [14], [15], [16], [17], [18], [19]]. While individual comorbidities are known to contribute to worse ablation outcomes, the role of multimorbidity, defined as the coexistence of two or more chronic conditions in the same individual, on atrial arrhythmia recurrence remains unclear. Therefore, this study aimed to investigate the association between multimorbidity and atrial arrhythmia recurrence after cryoballoon AF ablation.

## 2 Methods

### 2.1 Study design

The cryoballoon ablation study is a prospective, single-center registry aimed at identifying a risk profile associated with failure of the cryoballoon AF ablation procedure. Patients were eligible if they consented to the cryoballoon AF ablation procedure and were over 18 years of age. Those with a history of prior AF ablation were excluded. A total of 349 consecutive patients who received their first cryoballoon AF ablation at the University Medical Center Groningen (UMCG), the Netherlands, between October 2018 and July 2022 were included. Baseline assessments for all patients included clinical history, current medication use, physical examination, blood sampling, electrocardiogram (ECG), 24-hour Holter monitoring, echocardiography, and computed tomography imaging of the heart. Routine clinical follow-up visits, combined with ECG recording and 24-hour Holter monitoring, were scheduled at 3, 6, and 12 months after the procedure to capture the outcome of recurrence. The study was approved by the institutional medical Ethics Committee and performed in concordance with the Declaration of Helsinki. All patients gave written informed consent.

### 2.2 Cryoballoon AF ablation procedure

The ablation procedure was performed with the patient awake, under conscious sedation, or under deep sedation. Left atrium access was achieved with a single transseptal puncture, followed by the administration of an intravenous bolus of heparin at 70–100 IU/kg. A doubled walled cryoballoon (Artic Front Advance, Cryocath), either 23 or 28 mm depending on the pulmonary vein ostium size and physician preference, was used for ablation. Per pulmonary vein one or two consecutive applications with a maximum duration of 180 s was delivered. The number of applications was limited to one if time to isolation was less than 60 s. A second application was delivered if the time to isolation exceeded 60 s. Usually, the left superior pulmonary vein was treated first, followed by the left inferior pulmonary vein, right inferior pulmonary vein, and right superior pulmonary vein. During cryothermal ablation of the right sided veins, diaphragmatic stimulation, using a decapolar catheter placed in the superior caval vein, was performed to avoid phrenic nerve injury. Electrical isolation of the pulmonary veins was evaluated using the circular Achieve mapping catheter (entrance and exit block).

### 2.3 Definitions of comorbidities and multimorbidity

Multimorbidity was defined as the presence of at least two of the following comorbidities: hypertension, hypercholesterolemia, heart failure, coronary artery disease, diabetes mellitus, obesity, renal dysfunction, thyroid dysfunction, previous transient ischemic attack (TIA) or stroke, peripheral artery disease (PAD) and chronic obstructive pulmonary disease (COPD). Hypertension was defined as systolic blood pressure ≥ 140 mm Hg or diastolic blood pressure ≥ 90 mm Hg, or use of antihypertensive medication. Hypercholesterolemia was defined as total serum cholesterol > 6.5 mmol/L or use of cholesterol-lowering medication. Heart failure was defined as a history of heart failure admission or left ventricular ejection fraction (LVEF) ≤ 45 %. Diabetes mellitus was defined as a documented diagnosis of diabetes mellitus or use of antidiabetic medication. Coronary artery disease was defined as any of the following; significant coronary artery stenosis confirmed by coronary angiography, myocardial infarction, previous percutaneous intervention, and/or coronary artery bypass grafting. Obesity was defined as a body mass index > 30 kg/m2. Renal dysfunction was defined as an estimated glomerular filtration rate (eGFR) < 60 ml/min/1.73 m2. Thyroid dysfunction was defined as serum thyroid-stimulating hormone levels < 0.5 or > 4 mU/L or the use of thyroid medications. Diagnoses of previous TIA/stroke, COPD, and PAD were all obtained from the patient's history recorded in the electronic patient files.

### 2.4 Outcomes

The outcome was recurrence of atrial arrhythmia within one year after cryoballoon AF ablation. Recurrence of atrial arrhythmia was defined as any episode of AF, atrial flutter, or atrial tachycardia lasting longer than 30 s documented on 12-lead ECG, 24-hour Holter monitoring, event recorder or implantable loop recorder. The blanking period was defined as the first eight weeks following the ablation procedure [3].

### 2.5 Statistical analysis

Descriptive statistics for continuous variables are presented as mean ± standard deviation or median [interquartile range (IQR)], depending on normality of the data. Categorical variables are presented as numbers with percentages. To assess the association between the individual comorbidities, number of comorbidities, multimorbidity and atrial arrhythmia recurrence, univariate Cox proportional hazards regression analyses were conducted. Age and sex were included in the multivariable model for adjustment. The proportionality of hazards assumption was confirmed for each model. Kaplan-Meier estimates for the cumulative survival of sinus rhythm within one year after the ablation procedure were calculated and plotted, with the log-rank test used to compare the groups. All statistical analyses were conducted using IBM SPSS Statistics for Windows, version 28.0 (IBM Corp., Armonk, NY). Two-sided p-values < 0.05 were considered statistically significant.

## 3 Results

### 3.1 Patient characteristics

**Fig. 1:**
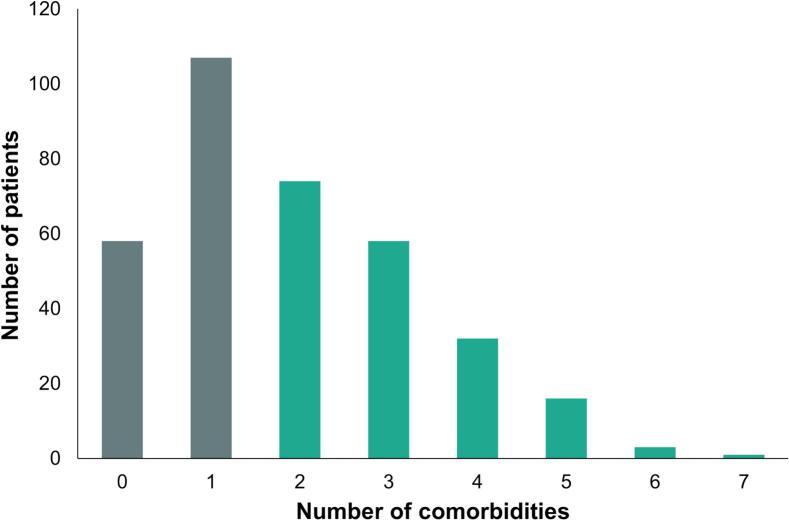
Number of comorbidities in the study population.

**Fig. 2:**
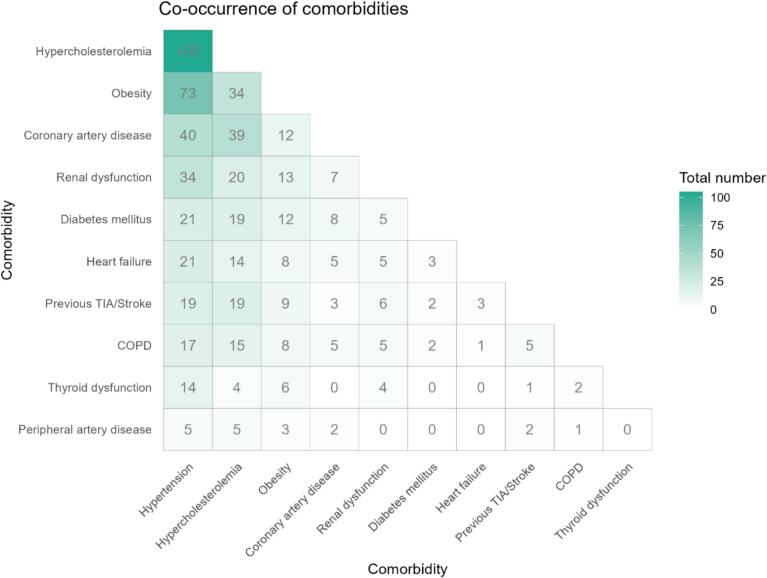
Overview of comorbidity combinations in the study population.

Baseline characteristics of the study population are presented in Table 1. In total, we analysed 349 patients with a mean age of 62 ± 9 years and 123 (35 %) women. Of these 349 patients, 199 (57 %) had paroxysmal AF, 148 (42 %) persistent AF, and 2 (1 %) longstanding persistent AF. The median number of comorbidities was 2 [1–3] and 184 (53 %) patients had multimorbidity. Fig. 1 illustrates how comorbidities were distributed across the population. The most prevalent comorbidities were hypertension (71 %), hypercholesterolemia (34 %) and obesity (25 %). An overview of the comorbidity combinations within this population is presented in Fig. 2, showing that the most frequent combinations are hypertension with hypercholesterolemia (105 patients (30 %)), hypertension with obesity (73 patients (21 %)), and hypertension with coronary artery disease (40 patients (11 %)). During a median follow-up of 328 [IQR 203–409] days, 114 patients (33 %) experienced an atrial arrhythmia recurrence within one year after cryoballoon AF ablation. Among them, 97 (85 %) had AF, 11 (10 %) had atrial flutter, and 6 (5 %) had atrial tachycardia.

**Table 1:** Patient characteristics.

**Characteristic**	
Age (years)	62 ± 9
Female sex	123 (35 %)
*Type of AF*	
Paroxysmal AF	199 (57 %)
Persistent AF	148 (42 %)
Longstanding persistent AF	2 (1 %)
Time since first AF diagnosis (months)	33 [11–––89]
BMI (kg/m^2^)	27.6 ± 4.4
Number of comorbidities	2 [[1], [2], [3]]
0-1	165 (47 %)
≥ 2	184 (53 %)
*Comorbidities*	
Heart failure	25 (7 %)
Hypertension	248 (71 %)
Hypercholesterolemia	119 (34 %)
Diabetes mellitus	23 (7 %)
Obesity	88 (25 %)
Coronary artery disease	46 (13 %)
Previous TIA/stroke	30 (9 %)
Peripheral artery disease	6 (2 %)
Renal dysfunction	41 (12 %)
COPD	20 (6 %)
Thyroid dysfunction	16 (5 %)
*Medication at baseline*	
Beta-blocker	161 (46 %)
Digoxin	15 (4 %)
Calcium channel blocker	110 (32 %)
AAD I	105 (30 %)
AAD III	91 (26 %)
ACE-I/ARB	138 (40 %)
Diuretics	54 (15 %)
Anticoagulant	341 (98 %)
Statin	109 (31 %)

AF = atrial fibrillation; BMI = body mass index; TIA = transient ischemic attack; COPD = chronic obstructive pulmonary disease; AAD I = anti-arrhythmic drugs class I; AAD III = anti-arrhythmic drugs class III; ACE-I = angiotensin converting enzyme inhibitor; ARB = angiotensin receptor blocker.

### 3.2 Individual comorbidities and arrhythmia recurrence after ablation

**Fig. 3:**
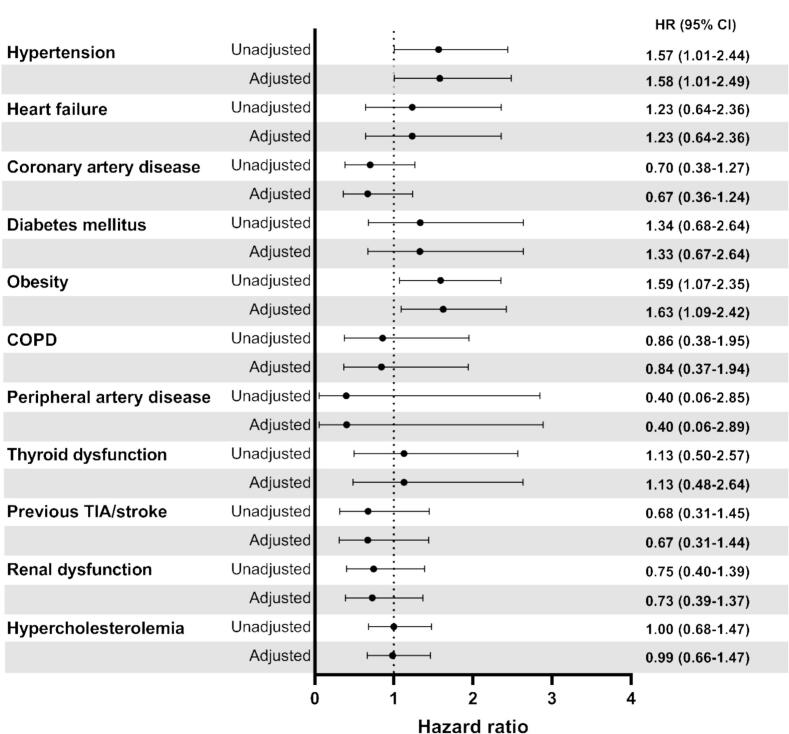
Forest plot of the individual comorbidities and risk of atrial arrhythmia recurrence within 1 year following cryoballoon AF ablation.

Fig. 3 shows the association between the individual comorbidities and recurrence of AF. Cox proportional hazard regression analyses for each individual comorbidity revealed that hypertension (HR 1.57, 95 % CI (1.01–2.44), p = 0.047) and obesity (HR 1.59, 95 % CI (1.07–2.35), p = 0.021) were associated with atrial arrhythmia recurrence within one year after cryoballoon AF ablation. These associations remained statistically significant after adjustments for age and sex (HR 1.58, 95 % CI (1.01–2.49), p = 0.047 and HR 1.63, 95 % CI (1.09–2.42), p = 0.017, respectively). No associations were found between the other individual comorbidities and the outcome.

### 3.3 Multimorbidity and arrhythmia recurrence after ablation

**Fig. 4:**
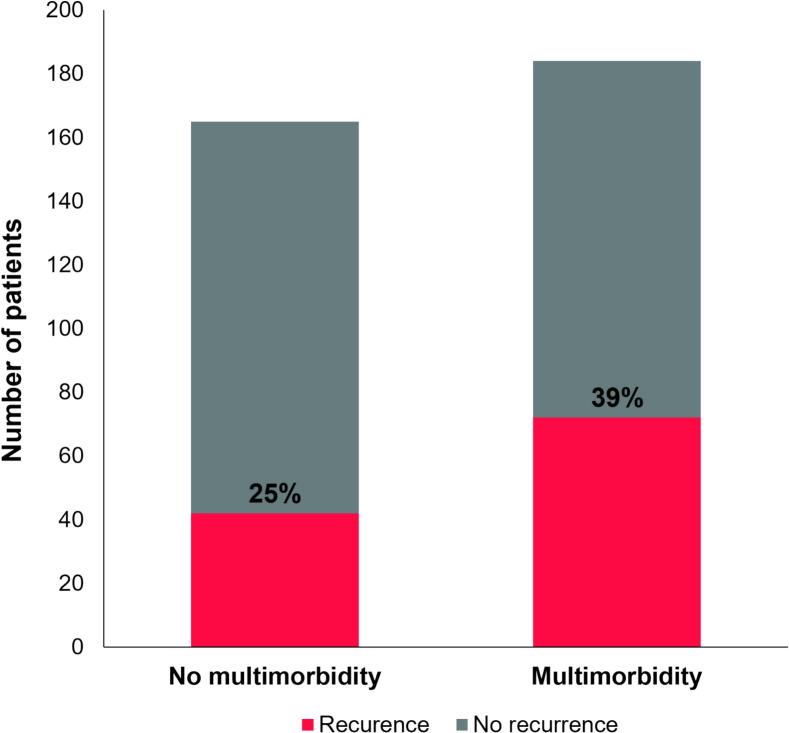
Multimorbidity and atrial arrhythmia recurrence.

**Fig. 5:**
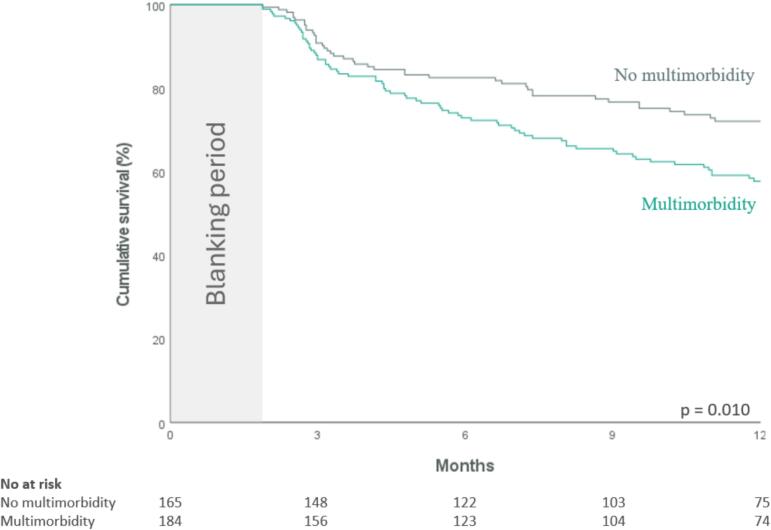
Kaplan-Meier curve for the cumulative atrial arrhythmia-free survival within 1 year following cryoballoon AF ablation according to the presence of multimorbidity.

In patients with multimorbidity, 72 (39 %) experienced recurrence, compared to 42 (25 %) in those without multimorbidity (Fig. 4). Cox proportional hazard regression analysis showed that multimorbidity was significantly associated with arrhythmia recurrence within one year following cryoballoon AF ablation (HR 1.64, 95 % CI (1.12–2.39), p = 0.011). After adjustments for age and sex, this association remained significant (HR 1.66, 95 % CI (1.12–2.45), p = 0.011). The cumulative atrial arrhythmia-free survival throughout the follow-up period is illustrated by the Kaplan-Meier curve in Fig. 5. However, when we performed the Cox regression analysis with the number of comorbidities as a continuous variable, we did not observe a significant association with atrial arrhythmia recurrence after cryoballoon ablation (HR 1.05, 95 % CI (0.93–1.19), p = 0.451).

## 4 Discussion

In the present study, multimorbidity was present in about half of the patients, and those with multimorbidity had an increased risk of atrial arrhythmia recurrence. Additionally, we identified hypertension and obesity as individual comorbidities independently associated with atrial arrhythmia recurrence within one year after cryoballoon AF ablation.

### 4.1 Multimorbidity and arrhythmia recurrence after ablation

Our research findings indicate that multimorbidity is significantly associated with an increased risk of atrial arrhythmia recurrence within one year following cryoballoon AF ablation. Furthermore, in this patient population, the most frequent combinations of comorbidities were hypertension with hypercholesterolemia, hypertension with obesity, and hypertension with coronary artery disease.

Previous research has shown that multimorbidity increases the likelihood of developing AF [20,21] and, in those already diagnosed with AF, contributes to disease progression [22] and adverse clinical outcomes. [[23], [24], [25], [26]] However, data concerning the impact of multimorbidity on ablation outcomes is limited. A relevant study in this area is a sub-analysis of the ESC-EHRA EORP registry, which examined the influence of various risk factors, mainly consisting of comorbidities, on the recurrence of AF following catheter ablation. [27] The authors categorized patients into two groups based on risk factors: those with one or more risk factors and those without. Results indicated that having at least one risk factor increased the risk of arrhythmia recurrence after ablation by 30 % compared to patients with no risk factors. While multimorbidity is not specifically addressed in this study, the comparison still provides valuable information. Looking at comorbidities in a grouped way, rather than focusing on a single condition, allows for a more comprehensive view of risk factors and captures a broader risk profile that individual comorbidities alone may not reveal. Additionally, since most AF patients have more than one comorbidity [28] this approach better represents the actual patient population that is seen in daily clinical practice. Building upon this, Silva Cunha et al. found that AF patients with more than one comorbidity experienced an increased recurrence risk after ablation compared to patients with no or one comorbidity, however this was only significant beyond approximately 30 months of follow-up after catheter ablation. [29] While studies evaluating the effect of multimorbidity on recurrence risk after ablation are limited, research has been conducted on the benefits of addressing risk factors before ablation. In particular, the ARREST-AF trial provided important insights into the benefits of risk factor modification in patients undergoing initial AF catheter ablation. [30] Patients were either assigned to a dedicated clinic for structured risk factor management (intervention) or given informational guidance and continued with their physician's usual management (control). Risk factor management included blood pressure control, weight and lipid management, glycemic control, sleep-disordered breathing management, and attempt at smoking cessation and alcohol reduction. The results showed a greater reduction in AF frequency, duration, symptoms, and symptom severity in the group that received risk factor management. Furthermore, a higher percentage of patients remained free from atrial arrhythmia in the two years following AF ablation in the group that received risk factor management. In addition, a retrospective study by Donnelan et al. demonstrated that arrhythmia-free survival rates were linked to the number of risk factors managed before ablation, including sleep apnea screening and management, glycemic control, blood pressure management, and weight loss. Higher survival rates were observed when more risk factors were successfully modified. [31].

### 4.2 Individual comorbidities and arrhythmia recurrence after ablation

In our study, we also identified individual comorbidities to be associated with atrial arrhythmia recurrence within one year after cryoballoon AF ablation. The presence of hypertension was associated with a 58 % higher risk of atrial arrhythmia recurrence following cryoballoon AF ablation. This finding aligns with the current literature, which highlights that hypertension significantly influences AF ablation outcomes, especially when hypertension is inadequately managed [[5], [6], [7], [8], [9], [10]]. Parkash et al. further explored the relation between hypertension and arrhythmia recurrence with a randomized controlled trial comparing blood pressure targets of < 120 mmHg and < 140 mmHg in paroxysmal and persistent AF patients undergoing ablation. Interestingly, they found that aggressive blood pressure management did not reduce arrhythmia recurrence compared to standard treatment [32]. The absence of a significant effect may be attributed to the limited sample size and modest blood pressure differences between groups. A novel approach for managing blood pressure as secondary prevention of AF involves renal denervation during AF ablation. Pokushalov et al. conducted a randomized controlled trial with patients having paroxysmal or persistent AF, assigning them to receive either PVI alone or in combination with renal artery denervation. The study demonstrated that those undergoing additional renal denervation had significant reductions in both systolic and diastolic blood pressure, along with fewer AF recurrences compared to those only receiving PVI [33]. Similarly, the ERADICATE-AF study indicated that paroxysmal AF patients with hypertension who underwent catheter ablation along with renal denervation had a reduction in systolic blood pressure and experienced a greater reduction in atrial arrhythmias compared to those who received only catheter ablation [34].

Our study also revealed an association between obesity and outcomes after cryoballoon AF ablation, with patients with obesity having a 63 % higher risk of atrial arrhythmia recurrence. This association has also been demonstrated in previous research [[11], [12], [13]]. Additionally, weight loss has shown beneficial effects on arrhythmia-free survival in AF patients in the LEGACY observational study [35]. For the purpose of this study, weight loss was categorized into three groups: ≥10 %, 3 % to 9 %, and < 3 %. The investigators found that the group with ≥ 10 % weight loss had six times greater arrhythmia-free survival than the other groups. However, this study did not focus solely on arrhythmia-free survival after ablation, including patients with and without rhythm control strategies. The SORT-AF trial, in contrast, specifically examined outcomes after ablation in patients with paroxysmal and persistent AF who were randomized to either weight reduction or usual care following the procedure. The results showed a significant reduction in body mass index in de intervention group compared to the usual care group, however it did not impact ablation outcomes [36]. A possible explanation for the lack of observed impact could be the average weight loss, which was only about 4 %.

In our exploration of comorbidities associated with recurrent AF after cryoballoon ablation, we found no significant associations with hypercholesterolemia, heart failure, coronary artery disease, diabetes mellitus, renal dysfunction, thyroid dysfunction, previous TIA/stroke, PAD, or COPD. Despite our findings showing no association, previous studies consistently link comorbidities like diabetes mellitus [[14], [15], [16], [17]] and heart failure [18] to arrhythmia recurrence after ablation. The absence of a significant correlation in our study might be attributed to the relatively modest sample size and the characteristics of our study population, which was largely healthy with few comorbidities.

### 4.3 Clinical implications

Our findings demonstrate that multimorbidity is linked to atrial arrhythmia recurrence after ablation, while other studies have shown that risk factor management can improve outcomes post-ablation. For clinical practice, this indicates the importance of identifying and optimizing comorbidities prior to AF ablation to improve patient outcomes. However, further research is needed to identify which comorbidities most significantly impact ablation outcomes and to determine the best order to address comorbidities when multiple are present. Identifying the most influential comorbidities could guide clinicians in focusing on factors that have the greatest effect on ablation outcomes or are the most feasible to address in the short term. Additionally, understanding the threshold at which a comorbidity is considered “sufficiently managed” for ablation remains a key question. Furthermore, we did not observe a significant association between the number of comorbidities, treated as a continuous variable, and atrial arrhythmia recurrence after ablation. This may suggest that there is no clear trend toward a higher recurrence rate with an increasing number of comorbidities. However, this finding could also be attributed to the modest sample size of our study. Overall, further studies are required, but their influence on daily practice will be considerable.

## 5 Limitations

Albeit that this prospective observational cohort study on PVI using cryoballoon provides insights into the association between multimorbidity and atrial arrhythmia recurrence, some limitations need consideration. The absence of continuous rhythm monitoring may have resulted in missed episodes of both symptomatic and asymptomatic atrial arrhythmia recurrence. Also, there was no information available on the severity of most comorbidities. The comorbidities were therefore included as dichotomous variables, failing to account for the impact of varying severity on ablation outcomes. The relatively modest sample size limited the ability to perform subgroup analyses in this study. Furthermore, we cannot exclude residual confounding. The extent to which our findings can be extrapolated to ablation cohorts employing other ablation modalities, such as radiofrequency ablation or pulsed field ablation, remains uncertain.

## 6 Conclusions

In AF patients undergoing cryoballoon AF ablation, multimorbidity was associated with atrial arrhythmia recurrence within one year post-ablation. Additionally, we found that hypertension and obesity were the only individual comorbidities independently associated with the outcome. Further research is needed to determine the best treatment approach for patients with multimorbidity to improve their chances of a successful ablation.

## 7 Disclosures

M.R. reports consultancy fees from Bayer (OCEANIC-AF national PI) and InCarda Therapeutics (RESTORE-1 national PI) to the institution. M.R. reports an unrestricted research grant from the Dutch Heart Foundation and is conducted in collaboration with and supported by the Dutch CardioVascular Alliance, 01–002-2022–0118 EmbRACE. Unrestricted research grant from ZonMW and the Dutch Heart Foundation; DECISION project 848090001. Unrestricted research grants from the Netherlands Cardiovascular Research Initiative: an initiative with the support of the Dutch Heart Foundation; RACE V (CVON 2014–9), RED-CVD (CVON2017-11). Unrestricted research grant from Top Sector Life Sciences and Health to the Dutch Heart Foundation [PPP Allowance; CVON-AI (2018B017)]. Y.B. reports receiving Research grant from Boston Scientific.

## CRediT authorship contribution statement

**Marieke J.H. Velt:** Visualization. **Colinda van Deutekom:** Visualization. **Michelle Lobeek:** Writing – review & editing, Visualization. **Michiel Rienstra:** Writing – review & editing, Conceptualization. **Yuri Blaauw:** Writing – review & editing, Funding acquisition. **Bart A. Mulder:** Visualization.

## Funding

The cryoballoon registry was supported by a research grant from Medtronic. C.v.D. and M.R. reports an unrestricted grant from the European Union’s Horizon 2020 research and innovation programme under grant agreement; EHRA-PATHS (945260).

## Declaration of competing interest

The authors declare the following financial interests/personal relationships which may be considered as potential competing interests: [The cryoballoon registry reports financial support was provided by Medtronic Inc. Colinda van Deutekom reports financial support was provided by Horizon Europe. Michiel Rienstra reports financial support was provided by Horizon Europe. Michiel Rienstra reports a relationship with Bayer AG that includes: consulting or advisory. Michiel Rienstra reports a relationship with InCarda Therapeutics, Inc. that includes: consulting or advisory. Michiel Rienstra reports a relationship with Netherlands Heart Foundation that includes: funding grants. Michiel Rienstra reports a relationship with ZonMW that includes: funding grants. Yuri Blaauw reports a relationship with Boston Scientific Corporation that includes: funding grants. If there are other authors, they declare that they have no known competing financial interests or personal relationships that could have appeared to influence the work reported in this paper.].
